# Zooming in: PAGE-Northern Blot Helps to Analyze Anti-Sense Transcripts Originating from Human rIGS under Transcriptional Stress

**DOI:** 10.3390/ncrna7030050

**Published:** 2021-08-24

**Authors:** Anastasia A. Sadova, Dmitry Y. Panteleev, Galina V. Pavlova

**Affiliations:** 1 Academic Chair of Biochemistry and Molecular Biology, Faculty of General Medicine, Pirogov Russian National Research Medical University, 117997 Moscow, Russia; 2 Institute of Biomedical Problems, Russian Academy of Sciences, 123007 Moscow, Russia; 3 Institute of Higher Nervous Activity and Neurophysiology, Russian Academy of Sciences, 117485 Moscow, Russia; mycobiota@yandex.ru (D.Y.P.); lkorochkin@mail.ru (G.V.P.); 4 Institute of Molecular Medicine, Sechenov First Moscow State Medical University, 119991 Moscow, Russia; 5 Department of X-ray and Radioisotope Diagnostic Methods, Burdenko Neurosurgical Institute, 125047 Moscow, Russia

**Keywords:** northern blot, non-coding RNA, small RNA, rRNA, rIGS, rRNA depletion

## Abstract

Ribosomal intergenic spacer (rIGS), located between the 45S rRNA coding arrays in humans, is a deep, unexplored source of small and long non-coding RNA molecules transcribed in certain conditions to help a cell generate a stress response, pass through a differentiation state or fine tune the functioning of the nucleolus as a ribosome biogenesis center of the cell. Many of the non-coding transcripts originating from the rIGS are not characterized to date. Here, we confirm the transcriptional activity of the region laying a 2 kb upstream of the rRNA promoter, and demonstrate its altered expression under transcriptional stress, induced by a wide range of known transcription inhibitors. We managed to show an increased variability of anti-sense transcripts in alpha-amanitin treated cells by applying the low-molecular RNA fraction extracted from agarose gel to PAGE-northern. Also, the fractioning of RNA by size using agarose gel slices occurred, being applicable for determining the sizes of target transcripts via RT-PCR.

## 1. Introduction

Recently, much attention has been drawn to the investigation of small non-coding RNA molecules, originating from different parts of the genome, which possess a myriad of diverse functions in living creatures [1,2]. Many researchers make use of contemporary approaches for the characterization of these transcripts such as bioinformatic analysis of various RNA-seq databases, and computer modelling based on already existing information on multiple classes of non-coding RNAs in different species [3,4]. This “dry-biology” data must be verified by in-lab experimental procedures. The traditionally used “wet-biology” northern blot analysis is a handy technique allowing the detection and characterization of RNA transcripts. Although radioactive isotopes—widely used before—make it applicable for the analysis of transcripts present in cells in extremely low amounts, many laboratories are restricted to the use of less-hazardous biochemically labeled RNA probes (e.g., biotinylated probes, digoxigenin-labeled probes, fluorescent and amine labeling [5,6,7], which are not so sensitive, and the minor RNA molecules remain concealed [8]. Another problem faced by many scientists working or eager to work with RNAs—especially small RNAs—is the extraction of the molecules from gels after their separation using electrophoresis. The kits and reagents proposed commercially are optimized for DNA, but not for RNA. There exist several protocols allowing the extraction of RNA from agarose and polyacrylamide gels using diffusion or electroelution [9,10,11]; however, the process can be further improved, as it is primarily applicable for the RNA molecules abundant in samples such as ribosomal RNAs or major transcripts of actively expressed genes. Such complications are challenging for those who explore non-coding regions of the genome, which can give rise to a variety of small and long RNA transcripts of yet unknown functions transcribed in cells under specific conditions, such as stress.

**Figure 1:**
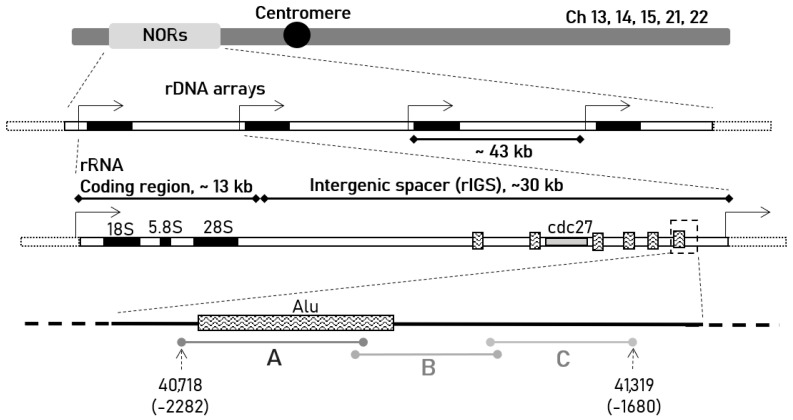
The schematic representation of human rDNA arrays located in the nucleolar-organizing regions (NORs) on short arms of acrocentric chromosomes, each containing ~13 kb coding region for 18S, 5.8S and 28S rRNAs, and ~30 kb ribosomal intergenic spacer (rIGS). Features in the rIGS such as Alu-repeats and cdc27 pseudogene are schematically represented based on the available data [15]. The region under investigation in the current work is located ~2 kb upstream of the rDNA transcription start site (black arrows) and highlighted by dotted-line arrows indicating the positions of nucleotides (according to GenBank sequence U13369.1, https://www.ncbi.nlm.nih.gov/nuccore/555853 accessed on 1 June 2021). A, B and C segments represent the primer pairs designed to analyze transcriptional activity of the corresponding zone. The sequences are given in the Table A1.

In the current work we investigate the phenomenon of the transcription in the pre-promoter region of human ribosomal intergenic spacer (rIGS), which lies between rRNA-coding genes located in the nucleolar-organizing regions (NORs) in human acrocentric chromosomes [12,13] (Figure 1). Although the spacer itself is considered non-coding and not conserved in mammals and other animals compared to rRNA-coding regions [14], many recent studies demonstrate that it contains sequences, associated with or reminiscent of the starting points of transcription, and is actively transcribed [15]. In human lung cancer cells, the molecules originating from the rIGS help to immobilize proteins under acidification [16]. The pre-promoter region gives rise to a microRNA transcribed in certain human tissues [17]. In mice the zone near the rRNA promoter is transcribed resulting in a so-called promoter RNA, which regulates rRNA transcription and heterochromatin formation [18]. The existence of the RNA molecule in humans and the possibility of transcription of the corresponding region was also predicted [19]. It is demonstrated that the pre-promoter region of human rDNA is transcribed in cancer cells [20]. We also have shown that the region ~2 kb upstream of the rRNA promoter is transcriptionally active in normal human cell lines, as well as in glioma and glioblastoma cell cultures [21].

Little is known about the mechanisms of transcription regulation of the various non-coding RNAs originating from different parts of the rIGS, and the data to date does not clarify this question. RNA polymerase I (pol I), widely known to be associated with the nucleolus and rRNA-coding regions, was shown to be absent in the intergenic spacers of ribosomal genes [22]. On the contrary, RNA polymerase II (pol II) transcriptional activity was demonstrated in the non-coding regions of rDNA [23,24]. In addition, the rIGS contain multiple Alu-repeats, which are considered to be pseudogenes originating from RNA polymerase III (pol III) transcribed genes [25,26]. Therefore, the question of whether the ribosomal intergenic spacer is transcribed by pol I, II, or III needs elucidation, as well as the investigations of the mechanisms of the spacer’s transcriptional activity regulation, are to be conducted to further extend our knowledge about the role of these repetitive sequences in cells, tissues, and the whole organism.

Here we confirm the presence of small transcripts originating from the pre-promoter region of rRNA genes in human cells with the modified northern blot technique. We also attempt to estimate the transcriptional activity of the region under the influence of the RNA polymerase II inhibitor, alpha-amanitin, and evaluate the difference between the transcripts revealed in treated and non-treated cells. In addition, we try to assess the length of a possible precursor transcript by extracting the fractions of RNA molecules of different lengths from agarose gel.

## 2. Results

**Figure 2:**
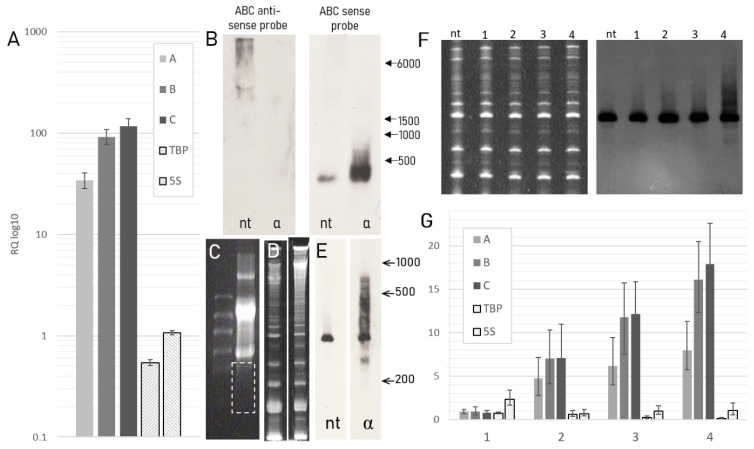
Gene expression analysis of alpha-amanitin treated cells versus non-treated HEK293 cells. (**A**) Relative quantity (log10) of A, B, and C regions, TBP and 5S after alpha-amanitin treatment (30 µg/mL) normalized to 18S and to non-treated cells as reference sample (ΔΔC_T_ method); (**B**) Northern blot analysis of total RNA from non-treated cells (nt) and cells treated with 30 µg/mL alpha-amanitin (α) with ABC anti-sense and sense RNA probes; (**C**) Agarose gel electrophoresis of total RNA showing the area extracted for high-resolution northern blot; (**D**) PAGE-northern blot of the extracted low-molecular weight RNA fractions from non-treated (nt) and alpha-amanitin treated (α) cells with ABC RNA probe; (**E**) PAGE of the extracted low-molecular weight RNA fractions; (**F**) PAGE and northern blot analysis in non-treated cells (nt) and cells treated with alpha-amanitin in the following concentrations: 1.5 µg/mL (1) 5 µg/mL (2) 10 µg/mL (3) 15 µg/mL (4) with ABC RNA probe; (**G**) Relative quantity of A, B, and C regions, TBP and 5S after alpha-amanitin treatment in the above mentioned concentrations normalized to 18S and to non-treated cells as reference sample (ΔΔC_T_ method).

We were eager to know whether the anti-sense transcripts, which we have recently demonstrated to originate from the zone ~2000 bp upstream of the rDNA promoter [21] (Figure 1), are transcribed by RNA polymerase II. To answer this question, we applied alpha-amanitin, known to inhibit eukaryotic RNA polymerases, primarily, pol II in the concentration of 30 µg/mL. Surprisingly, quantitative PCR analysis demonstrated a dramatic increase in the expression of the regions of interest after alpha-amanitin treatment compared to non-treated cells. The region A expression level increased only 30-fold, while B and C regions showed almost 100-fold increase in the expression after the exposure to the pol II inhibitor (Figure 2A). Next, we performed a northern blot analysis, which also confirmed the increased amount of target transcripts, which presumably result from an enhanced transcriptional activity of the zone under investigation in alpha-amanitin treated cells compared to non-treated cells (Figure 2B). We were motivated to obtain a higher-resolution image of the transcripts revealed by northern blot analysis in order to estimate the approximate size of the molecules expressed in the ABC region, which appeared upon alpha-amanitin treatment. A “manual” depletion of rRNA was performed (Figure 2C), and the subsequent PAGE-northern blot of the extracted RNA fraction allowed improvement of the image resolution (Figure 2D,E). This experiment revealed the presence of multiple transcripts of different lengths ranging from ~200 to ~800 nucleotides in the alpha-amanitin treated cells, with a major transcript of ~350 nucleotides shared by both treated and non-treated cells. It is important to note that the transcripts below the major one, concealed in the northern blot image performed traditionally, are clearly observed in the image obtained with the modified method. Interestingly, the effect of alpha-amanitin appeared to be dose dependent: both northern-blot analysis (Figure 2F) and real-time PCR (Figure 2G) demonstrated that the quantity of the target transcripts increased with the increasing of the concentration of the toxin in the media.

**Figure 3:**
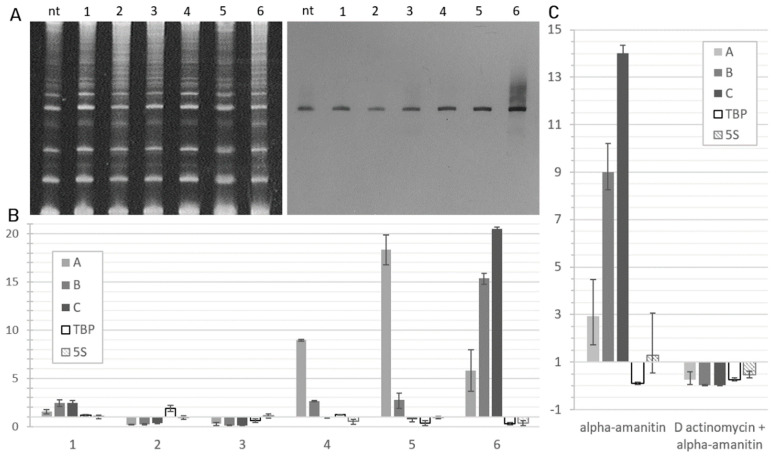
Gene expression analysis of HEK293 cells treated with transcription inhibitors: ML60218 (100 µM) (1) Belinostat (2 µM) (2) D actinomycin (40 ng/mL) (3) doxorubicin (4 µM) (4) BMH-21 (2 µM) (5) and alpha-amanitin (15 µg/mL) (6) compared to non-treated HEK293 cells (nt). (**A**) PAGE and northern-blot of low-molecular RNA fractions from non-treated and treated cells with the ABC sense probe. (**B**) Relative quantity of A, B, and C regions, TBP and 5S in HEK293 cells after the treatment with the indicated transcription inhibitors normalized to 18S and to non-treated cells as reference sample (ΔΔC_T_ method). (**C**) Relative quantity of A, B, and C regions, TBP and 5S in HEK293 cells after the treatment with alpha-amanitin (15 µg/mL) and combined alpha-amanitin and D actinomycin (15 µg/mL and 40 ng/mL, correspondingly) normalized to non-treated cells.

We tested other toxic substances known to halt DNA-dependent synthesis in the nuclei in an attempt to determine the enzyme providing the transcription of the region of interest and to analyze its expression in different transcriptional stress conditions. Even though the quantitative PCR analysis showed a heterogenous effect of the toxins on the transcription of the target sequences, none of the tested substances demonstrated the pattern observed in alpha-amanitin treated cells neither in RT-PCR, nor in PAGE-northern (Figure 3A). ML60218, proposed as an effective RNA pol III inhibitor [27], seemed to have no great effect on ABC expression (Figure 3B). Interestingly, belinostat, which is widely used in anti-cancer therapy as it induces apoptosis via the inhibition of histone deacetylases (HDAC), thus promoting excessive transcription in cells [28,29], led to a significant decrease in the expression of the region of interest. This effect was visualized by PAGE-northern, in which the amount of the target transcript was lower than in others, including non-treated control sample (Figure 3A). D actinomycin, which has been known for decades to inhibit transcription by interfering with the transcription complexes and RNA elongation [30], was recently demonstrated to block preferentially pol I mediated transcription when applied in low doses [31]. In our experiment, 40 ng/mL of D actinomycin decreased the expression of target sequences, though the effect was not clearly observed using PAGE-Northern. Other DNA-intercalating agents inducing transcriptional stress in cells used in the experiment were doxorubicin [32], and BMH-21, a specific pol I inhibitor [33], which both demonstrated the similar effect on the ABC expression. In doxorubicin treated cells as well as in BMH-21 treated cells the levels of the A region were 9 and 17 times higher correspondingly than in non-treated cells, and the levels of the B region increased 3-fold, while the expression of the C region was not affected. Northern-blot analysis demonstrated that bends in both doxorubicin and BMH-21 treated samples are nearly as sharp as in alpha-amanitin treated cells, though no clear smears of transcripts longer and shorter than the major one were observed, indicating that alpha amanitin has a unique impact on the expression of the target sequences. Interestingly, a combined treatment of cells with alpha-amanitin and D actinomycin led to a decrease in the expression of the target transcripts, which was demonstrated using RT-qRCP (Figure 3C).

**Figure 4:**
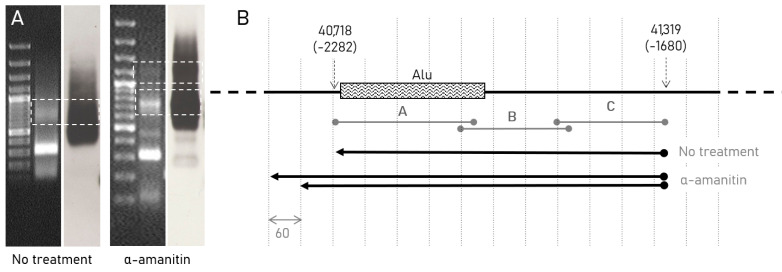
Mapping of the transcripts in the pre-promoter zone of rIGS. (**A**) Electrophoresis and Southern blot of cDNAs from non-treated and alpha-amanitin treated cells 30 µg/mL obtained with 3′RACE experiments, dotted rectangles showing the bands taken for cloning and subsequent sequencing; (**B**) Mapping of the sequences obtained from 3′ RACEs to the region of interest. Sequences and their pairwise alignments are available in the supplementary section (Supplementaries S1 and S2) or can be found using the GenBank accession numbers MW459196-MW459199.

Next, we applied 3′RACE to verify the results obtained by the northern blot. Southern blotting of the first-round RACE, obtained from the total RNA of the non-treated HEK293 cells, demonstrated bands lower than those of the first-round PCR with the cDNA, obtained from the total RNA of alpha-amanitin treated cells (Figure 4A). Cloning followed by the sequencing of the corresponding bands confirmed the fact that lengths of the transcripts predicted by the PAGE-northern blot analysis are different in treated and non-treated samples. In the alpha-amanitin treated cells we found several anti-sense transcripts of different lengths ranging from ~670 to 720 nucleotides, while in non-treated cells variants of 600–620 nucleotides were present (Figure 4B).

**Figure 5:**
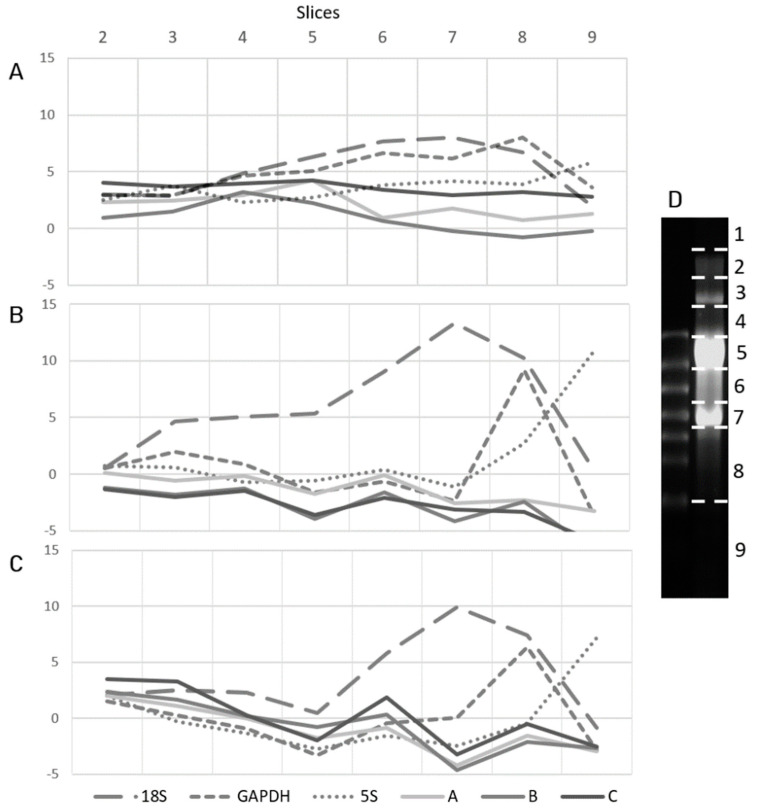
The preparation and analysis of RNA slices using RT-PCR. RT-PCR of slices obtained from (**A**) non-denaturing agarose gel electrophoresis of total RNA from non-treated cells, (**B**) denaturing agarose gel electrophoresis of total RNA from non-treated cells, (**C**) denaturing agarose gel electrophoresis of total RNA from alpha-amanitin treated cells (30 µg/mL) with the primer pairs for region of interest and reference genes; the graphs demonstrate the delta C_T_ values obtained for each of the primer pairs during the normalization of the C_T_ of each slice (2–9) to the C_T_ of slice 1 (ΔC_T_ = C_T_(1) − C_T_(2, 3, etc.). (**D**) Electrophoresis of non-denatured total RNA in 1% agarose gel with the schematic representation of sections made; slices 5 and 7 contain 28S and 18S rRNAs correspondingly.

The lengths of the transcripts obtained with RACEs, as well as the data of real-time PCR showing that the ABC region is transcribed as a whole, seem to be inconsistent with the results of northern blot analyses. The length of the major transcript, the presence of which was demonstrated with the latter method, is ~350 nucleotides, while the ABC zone being transcribed is ~600 bp, and, according to the results of step-out PCR, performed in order to determine the 3′-ends of the transcripts, it exceeds the bounds of the A region. In an attempt to determine the length of the transcript, which serves as template for reverse transcription and PCR, we separated total RNA from both alpha-amanitin treated (30 µg/mL) and non-treated HEK293 cells using non-denaturing and denaturing agarose gel electrophoresis and sliced the gel into 9 pieces each corresponding to a fraction of total RNA of different molecular weight and length (Figure 5D).

The subsequent RT-PCR analysis of RNA fractions with the primer pairs for the transcripts of known lengths such as 18S rRNA (1869 nt), GAPDH (1231–1525 nt), and 5S rRNA (121 nt) confirmed that we succeeded in separating the molecules according to their size. Even in non-denaturing conditions (Figure 5A) the slight peaks of expression of each of the targets appeared in the expected fractions, and in denaturing agarose gel the peaks appeared to be much sharper, indicating that denaturation of RNA by formamide and heat promoted the neat electrophoretic separation of the transcripts by length (Figure 5B,C).

As for the ABC region, we hoped to find the enrichment of its amplification among the low-size RNA molecules (fractions 8 and 9). However, in non-denaturing conditions no peak cycles were observed for A, B, and C primer pairs, except for a slight tendency to the fractions 1–5 of high molecular weight RNAs (≥5 kb, Figure 5A). The results of the experiment with the RNA fractions from alpha-amanitin treated cells were the same, except for the overall shifts in the threshold cycles caused by the treatment (not shown). We hypothesized that the cDNA being amplified by the primer pairs was synthesized from the transcript present in a small amount in all sizes of RNA fractions. On the other hand, the electrophoretic separation of the molecules was carried out under non-denaturing conditions, enabling the formation of RNA-secondary structures, RNA-RNA duplexes, and other complexes, which affect transcript mobility. Therefore, the same analyses were performed in denaturing conditions. In non-treated cells A, B, and C primer pairs tended to amplify cDNAs obtained from high-molecular weight RNA fractions (Figure 5B). The separation of total RNA from alpha-amanitin treated cells in denaturing conditions (Figure 5C) demonstrated two slight peaks for the studied primer pairs: the higher one in high-molecular weight RNA fractions and the lower one in the fraction 6 corresponding to transcripts ~2–4.5 kb in length. The overall pattern of expression of the regions of interest may be explained by the fact that they correspond to various RNA transcripts of different lengths rather than to a defined molecule.

To verify the result of the experiment, a two-factor ANOVA test was performed for each primer pair for denatured samples with one factor being the slice number, and the second factor being treatment. The analysis revealed the significant difference in the expression of GAPDH, 18S, 5S, and the region C between the treated and non-treated samples (*p* < 0. 05), while for primer pairs A and B the difference was not significant (*p* = 0.1 and *p* = 0.5 correspondingly). For all the primer pairs the analysis showed a significant difference in the expression between the slices (*p* < 0.05), so multiple comparison Tukey tests were applied to further analyze the difference between the slices. The comparison of the means of the slices for 5S demonstrated that slice 9 significantly differs from all the rest, what coincides with the observations obtained from the graph demonstrating the highest level of 5S expression in slice 9. Similar results were obtained for 18S and GAPDH with the expression of each tending to slices 7 and 8 correspondingly. The analysis of the means of the slices for the regions A, B, and C revealed the same pattern of significant difference for slices 5, 7, and 9 as the rest of the slices. On the graph the corresponding slices comply with the lowest expression of the regions of interest, which partially confirms the hypothesis that the original transcripts come from the RNA fractions of different molecular weight—primarily, the higher molecular weight. The calculations are attached as Supplementary S3.

## 3. Discussion

Ribosomal RNAs coding genes harbor intriguing features concerning the management of many of the cell’s functioning aspects [34], including heterochromatin formation [13], cell differentiation [35], protein synthesis [36], and stress response [37,38]. However, the main part of rDNA is non-coding, composing ribosomal intergenic spacers, which are poorly characterized in humans compared to other mammals.

In this study we confirm the transcriptional activity of the pre-promoter region of the human ribosomal intergenic spacer using northern blot and qPCR. Manual depletion of the transcripts > 1000 nucleotides, including 18S and 28S ribosomal RNAs, gave the opportunity to obtain high-resolution northern blot images after separation of the low-molecular weight RNA fraction using PAGE. The amount of the RNA material enriched in small RNAs and applied to the gel was thus increased, as PAGE is not applicable for large amounts (50–100 µg) of total RNA which overload the gel. The procedure of RNA extraction from agarose gel, reported in the study, was quite simple and handy, reminiscent of already existing protocols [9,10,11], but the use of extraction columns instead of diffusion and incubation at −20 °C instead of heating the samples, as recommended by other authors, ensures that the RNA remains suitable for subsequent manipulations, such as PAGE, reverse transcription and qPCR. The method of RNA extraction from agarose gel described here allows the isolation of virtually any of the desired RNA fractions, being ideal for the study of small RNA molecules. Compared to commercially available column-based methods for extraction of RNAs smaller than 200 nt, the technique proposed is relatively cheap and does not necessitate specific equipment or reagents except those traditionally present in a laboratory. As we demonstrated, agarose-extracted RNA fractions can be successfully reverse transcribed and amplified to determine the approximate length of a target transcript. The approach can be useful in RNA sequencing libraries and full-length cDNA libraries preparation.

Attempting to learn more about the phenomenon of rIGS transcription we applied a wide range of specific and non-specific transcription inhibitors; namely, ML60218, belinostat, D actinomycin, doxorubicin, BMH-21, and alpha-amanitin. The data obtained suggest that the inducement of transcriptional stress in cells has a differential effect on the expression of the pre-promoter region of human ribosomal intergenic spacer depending on the particular inducer. At the same time, the effect of alpha-amanitin observed in this study stands alone, indicating that the region of interest may possess unique functions during the transcriptional stress induced by specific RNA pol II inhibition.

The increased amount of target transcripts varying in length under the exposure to alpha-amanitin was demonstrated using qPCR and a modified technic of northern blot carried out after the extraction of low-molecular weight RNA from agarose gel and confirmed by 3′RACE experiments. Apparently, the anti-sense RNA molecules revealed are transcribed by either RNA pol I or RNA pol III, but not RNA pol II. Despite the fact that RNA pol II presence was demonstrated throughout the IGS, its specific enrichment tended to the regions IGS28 and IGS38 [24], while the region of interest in the current study is located in the IGS40-41. The study suggests that the ribosomal intergenic spacer is transcribed in the antisense orientation by RNA pol II, and not RNA pol I [24], which is not concordant with our results, as we showed the increased production of antisense transcripts after the inhibition of Pol II. On the other hand, our results demonstrate heterogenous effect of pol I inhibitors on the expression of the region under investigation, which may be explained by the different mechanisms of action of the toxins applied. However, the treatment of cells with D actinomycin together with alpha-amanitin restored the expression level of the target transcripts, supporting the hypothesis that the region of interest is transcribed by Pol I.

Additionally, according to the published data, the inhibition of RNA pol II can enhance the activity of pol III if the promoter of a gene is shared by the two enzymes [39,40]. Also, the region under investigation (ABC)—namely, its part designated A—contains an Alu-repeat. This type of SINE is hypothesized to originate from 7SL RNA known to be transcribed by pol III [41,42]. Interestingly, a nuclear isoform of mitochondrial RNA polymerase (spRNAP-IV) insensitive to alpha-amanitin was reported, and the expression of the genes transcribed by spRNAP-IV is either elevated [43] or remains unchanged after the treatment with the toxin [44].

The data of our previous investigations and current experiments results suggest at least two possible hypotheses able to explain the origin of the various antisense ABC transcripts (1) the molecules of different length are de novo transcribed in the conditions of transcriptional stress by an RNA polymerase insensitive to alpha-amanitin treatment, and changes in the regulation of transcription cause the different lengths of the transcripts (2) the transcripts originate from an unknown long non-coding RNA molecule, the processing of which is altered under alpha-amanitin stimulation. The qPCR and 3′RACE data partially belie the former idea, as the primers designed to cover 600 bp zone of interest apparently amplify a cDNA obtained on the template of a longer transcript. Therefore, the major ~350 nt transcript demonstrated with northern blot, as well as the minor shorter and longer molecules clearly revealed in PAGE-northern, that appeared after alpha-amanitin treatment of cells, are likely splice variants or processing variants originating from a long non-coding RNA molecule transcribed from the rIGS. This hypothesis is partially verified by the experiments with the fractioning of total RNA and subsequent RT–PCR analysis of the fractions of different molecular weight, in which we demonstrated a differential expression profile in alpha-amanitin treated and non-treated cells at least for the region C. However, northern blot analysis did not manage to demonstrate the precursor molecule, which thus remains concealed and claims further investigations with, possibly, some other methods capable of detecting such transcripts.

One more question to be elucidated is the type of non-coding RNAs which the transcripts revealed belong to. Despite the fact that further experiments aimed at characterization of the properties of the molecules are required, we propose that they are likely to belong to the class of long non-coding RNAs (lncRNAs). The transcripts under investigation are ~300 nt long and originate from intergenic regions transcribed in antisense orientation, what coincides with the features of lncRNAs, one of the largest and most heterogenous groups of ncRNAs. LncRNAs differ not only by size, but also by diverse posttranscriptional modifications (5′-cap or poly-A tail), complex secondary and tertiary structures, and various functions [45,46,47]. We plan to define the poly-A status of the target transcripts, to check if they belong to circular RNAs (circRNAs), to apply direct sequencing, and other experiments, which may further help to identify their biological roles.

## 4. Materials and Methods

### 4.1. Cell Cultures and Alpha-Amanitin Treatment

Prior to total RNA isolation HEK293 cell line originating from human embryonic kidney was grown in monolayer in a complete DMEM medium with 10% FCS at 37 °C, 98% humidity, 5% CO_2_. Cells were treated for 24 h with the indicated concentrations of alpha-amanitin or doxorubicin water solutions, or D actinomycin, ML60218, BMH-21, or Belinostat dissolved in DMSO. Untreated cells were grown in the medium containing the corresponding volume of water or DMSO as a vehicle.

### 4.2. RNA Extraction from Cells

RNA was extracted from cells using RNAzol RT reagent (MRC, Cincinnati, OH, USA) according to the manufacturer’s instructions for isolating total RNA with the subsequent treatment of samples with DNase I (Thermo Fisher Scientific, Vilnius, Lithuania) with the addition of RiboLock RNase Inhibitor (Thermo Fisher Scientific, Vilnius, Lithuania) for 40 min at 37 °C in an appropriate buffer solution. Following the DNase treatment, RNA samples underwent phenol-chloroform extraction, then were precipitated in isopropanol in the presence of 300 mM AcNa and washed with 75% (*w*/*v*) ethanol. Precipitates were dissolved in nuclease-free water and used in downstream analysis or stored at −70 °C. The purity of isolated RNA samples was tested using PCR analysis with 18S primers (Table A1).

### 4.3. Northern Blotting

About 10 µg of total RNA dissolved water with the addition of an appropriate volume of ×2 loading dye (Ambion, Carlsbad, CA, USA) was applied to denaturing gel electrophoresis in formaldehyde running buffer (1 M MOPS, 0.5 M sodium acetate, 50 mM EDTA, 2.2 M formaldehyde). Capillary transfer to positively charged nylon membrane (Roche, Mannheim, Germany) was performed in transfer buffer (3 M NaCl, 10 mM NaOH) for 2 h with the subsequent covalent linkage to the membrane by 254 nm UV light during 50 s. Biotinylated RNA probe was synthesized from a plasmid containing a corresponding sequence (Table A1) using T7 RNA Polymerase (Thermo Fisher Scientific, Carlsbad, CA, USA) and Biotin RNA labelling mix (Roche, Indianapolis, IN, USA) according to the manufacturer’s instructions.

Hybridization was performed in UltraHyb Ultrasensitive Hybridization Buffer (Invitrogen, Carlsbad, CA, USA) at 68 °C overnight, after that the membrane was washed in buffers A (0.1% SDS, 0.1× SSC) and B (20 mM Tris, 150 mM NaCl, 0.05% Tween-20 (*w*/*v*) and incubated in blocking solution (maleic buffer (100 mM Maleic acid, 150 mM NaCl, pH 7.5) containing 1% Blocking reagent (*w*/*v*) (Roche, Mannheim, Germany) for one hour at room temperature, with the subsequent addition of the appropriate amount of Pierce Streptavidin Poly-HRP to the blocking solution and incubation at room temperature for an hour. After washing in TBS-T, detection was performed using the Amersham ECL Prime Western Blotting Detection Reagent (Merck KGaA, Darmstadt, Germany) and developing proceeded by Amersham Hyperfilm ECL (Merck KGaA, Darmstadt, Germany).

### 4.4. Reverse Transcription and Quantitative PCR

Reverse transcription was performed in the reaction volume of 10 µL using MMLV RT kit (Evrogen, Moscow, Russia), 2 µg of total RNA was reverse transcribed using random hexamer primer and 100 units of MMLV RT at 42 °C for 60 min.

The comparative C_T_ experiment was performed on AB StepOnePlus Real-Time PCR system (Thermo Fisher Scientific, Carlsbad, CA, USA) using SYBR Green reagent qPCRmix-HS SYBR + HighROX (Evrogen, Moscow, Russia) with the reaction set up according to the manufacturer’s instructions. The reactions were performed in triplicates and included negative controls in which RNA was added instead of the template. The run protocol consisted of 40 cycles of 95 °C for 15 s, 60 °C for 15 s, and 72 °C for 30 s, with fluorescence intensity measurement performed after the elongation stage. The specificity of PCR products was confirmed using a melting curve analysis. The expression of target sequences in alpha-amanitin treated and non-treated cells was normalized to that of the 18S rRNA. Analysis of the fold difference in the expression of the regions of interest were performed with StepOne Software v2.3 (Thermo Fisher Scientific, USA). The primers for the regions of interest, 18S rRNA, GAPDH, and TBP are given in the Appendix A (Table A1).

### 4.5. rRNA Depletion and Slices Preparation

For the fractioning of transcriptome according to the size of the molecules 20–100 μg of total RNA with the addition of 6× Loading dye were loaded to 0.6% agarose gel electrophoresis in 1× TAE. Low-molecular fraction of RNA for further polyacrylamide northern-blot analysis was prepared by the extraction of the region under 1000 bp from the gel (see Figure 2D). For slices preparation 20 μg of total RNA was separated into 1% agarose gel and the whole lane was sectioned into 9 pieces (see Figure 5D). In case of denaturing agarose gel RNA samples were dissolved in 50% formamide with the addition of ×2 loading dye (Ambion, Carlsbad, CA, USA), heat-denatured at 65 °C for 10 min and cooled immediately, then applied to 1% agarose gel containing 1% of household bleach (final sodium hypochlorite concentration in the gel 0.05–0.15%) [48]. The pieces of gel were put into Pierce Spin Columns (ThermoFisher Scientific, Rockford, IL, USA) with pre-placed large frits and incubated at −20 °C for 1 h or more for agarose disruption. Then the columns with the samples were placed in 2 mL centrifuge tubes and centrifuged at 21,000× *g* at 4 °C. Filtrate was transferred to a sterile tube and proceeded by phenol-chloroform extraction and subsequent precipitation in isopropanol and washing with ethanol. Precipitants were dissolved in nuclease-free water and used in downstream analysis (PAGE northern blot or RT-PCR) or stored at −70 °C.

### 4.6. Statistics

Two-factor ANOVA test was performed using the Analysis ToolPak in Excel from Microsoft Office Professional Plus 2016 (version 2101). The subsequent multiple comparison Tukey HSD test was performed manually for each of the primer pairs, using the means for each of the slices. Critical ranges were obtained using the Equation (1):

(1) $$Critical\;range=q\surd \frac{MS}{n}$$

where *q* values were obtained from the studentized range *q* tables, *MS* were obtained from the ANOVA test, *n* was the number of observations. If the absolute difference exceeded the critical range, the difference between the slices was estimated as significant. The calculations are attached as a Supplementary S3.

### 4.7. PAGE Northern Blot

500 ng of the extracted RNA fraction was applied to 5% acrylamide gel containing 8 M Urea and 1× TBE, and electrophoresis was performed at 10 mA for 1.5 h. Next, we performed electroblotting to positively charged nylon membrane (Roche, Mannheim, Germany) at 400 mA in ice-cold 0.5× TBE for 30 min. Hybridization, membrane wash and development were the same as described in Section 4.3.

### 4.8. 3′RACE and Southern Blot

The first strand DNA synthesis was performed in 20 µL using ~2.5 μg of total RNA, a random hexamer primer and a 3′RACE adapter (Table A1) and 200 units of MMLV-RT (Evrogen, Moscow, Russia) in an appropriate buffer. Before the reaction the mixture of RNA, the primer and the adapter were denatured at 65 °C for 10 min and cooled on ice.

The reaction of the second strand synthesis was performed using Phusion High-Fidelity DNA Polymerase (Thermo Fisher Scientific, Carlsbad, CA, USA) in a volume of 20 μL according to the manufacturer’s instructions for reaction set up and 3-step run protocol. Cr primer (Table A1) was taken as gene specific primer. Aliquots of the first round PCR were analyzed using agarose gel electrophoresis and southern blotting. Membranes were incubated in Church-Gilbert buffer and hybridized with ABC biotinylated DNA probe (Table A1) at 63 °C overnight. Further steps of membrane washing, and detection procedure were as in the northern blot procedure described above. Following hybridization, we separated the samples by gel electrophoresis and the sharpest bends showing the highest signal were extracted, A-tailed, purified with phenol-chloroform and precipitated in isopropanol, and linked to the pGEM-Teasy vector. Plasmids from positive colonies were extracted and sequenced by Evrogen (Moscow, Russia). Sequence files and the alignment of the sequences are attached as supplementary files (Supplementaries S1 and S2 correspondingly).

## 5. Conclusions

In this study we report the phenomena of rIGS transcriptional activity in humans and an altered pattern of its transcription under alpha-amanitin treatment. The current investigation does not detail neither the processing of the transcripts revealed, nor their functions. Moreover, we are still unaware of the presence of any long anti-sense RNA molecule that could serve as a precursor of the 200–800 transcripts originating from the pre-promoter region of the rIGS. However, the data reported highlights the importance of further elucidation of the rDNA, and, especially, the functioning of ribosomal intergenic spacers in humans, in particular under stress conditions. The study also contains modified techniques and approaches that may be useful in the investigations of other non-coding still-transcribed regions, giving rise to small RNA transcripts.

## Data Availability

The sequences of non-coding transcripts under investigation obtained from RACE experiments were submitted to GenBank and can be found using the accession numbers MW459196-MW459199.

## Supplementary Materials

The following are available online at www.mdpi.com/2311-553X/7/3/50/s1, Supplementary S1: 3′RACE sequences from clones, Supplementary S2: Alignment of the sequences, Supplementary S3: Calculations for statistics.

## Appendix A

**Table A1:** The sequences of primers and probes.

Name	Sequence, 5′-3′	Amplicon Size, bp
A	GTTTCGTCCTTTTGAGACAGAGT, f GTGGGCGCATCACAGGAGGTC, r	254
B	TCCAACTCCCGACCTCCTGT, f TGCAGAGATACACGTTGTCGTTG, r	194
C	CAACGACAACGTGTATCTCTGCA, f TACGCTCGGTTCATTTACACACA, r	209
18S	TTTCTCGATTCCGTGGGTGG, f CCCGGACATCTAAGGGCATC, r	211
5S (from [49], modified)	GGCCATACCACCCTGAACG, f CAGCACCCGGTATTCCCAG, r	107
TBP	TCCCATGACCCCCATCACTC, f ATATTCGGCGTTTCGGGCA, r	145
GAPDH	ACCGTCAAGGCTGAGAACG, f GCATCGCCCCACTTGATTTT, r	95
3′RACE adapter	GCGAGCACAGAATTAATACGACTCACTATAGGT12VN	-
3′RACE primer	GCGAGCACAGAATTAATACGACT	
ABC sense RNA probe	GUUUCGUCCUUUUGAGACAGAGUUUCACUCUUGUUUCCACGGCUAGAGU GCAAUGGCGCGAUCUUGGCUCACCGCACCUUCCGCCUCCCGGGUUCGAGCGCUUCUCCUGCCUCCAGCCUCCCGAUUAGCGGGGAUUGACAGGGAGGCACCCCCACGCCUGGCUUGGCUGAUGUUUGUGUUUUUAGUAGGCACGCCGUGUCUCUCCAUGUUGCUCAGGCUGGUCUCCAACUCCCGACCUCCUGUGAUGCGCCCACCUCGGCCUCUCGAAGUGCUGGGAUGACGGGCGUGACGACCGUGCCCGGCCUGUUGACUCAUUUCGCUUUUUUAUUUCUUUCGUUUCCACGCGUUUACUUAUAUGUAUUAAUGUAAACGUUUCUGUACGCUUAUAUGCAAACAACGACAACGUGUAUCUCUGCAUUGAAUACUCUUGCGUAUGGUAAAUACGUAUCGGUUGUAUGGAAAUAGACUUCUGUAUGAUAGAUGUAGGUGUCUGUGUUAUACAAAUAAAUACACAUCGCUCUAUAAAGAAGGGAUCGUCGAUAAAGACGUUUAUUUUACGUAUGAAAAGCGUCGUAUUUAUGUGUGUAAAUGAACCGAGCGUA	
ABC anti-sense RNA probe	UACGCUCGGUUCAUUUACACACAUAAAUACGACGCUUUUCAUACGUAAAAUAAACGUCUUUAUCGACGAUCCCUUCUUUAUAGAGCGAUGUGUAUUUAUUUGUAUAACACAGACACCUACAUCUAUCAUACAGAAGUCUAUUUCCAUACAACCGAUACGUAUUUACCAUACGCAAGAGUAUUCAAUGCAGAGAUACACGUUGUCGUUGUUUGCAUAUAAGCGUACAGAAACGUUUACAUUAAUACAUAUAAGUAAACGCGUGGAAACGAAAGAAAUAAAAAAGCGAAAUGAGUCAACAGGCCGGGCACGGUCGUCACGCCCGUCAUCCCAGCACUUCGAGAGGCCGAGGUGGGCGCAUCACAGGAGGUCGGGAGUUGGAGACCAGCCUGAGCAACAUGGAGAGACACGGCGUGCCUACUAAAAACACAAACAUCAGCCAAGCCAGGCGUGGGGGUGCCUCCCUGUCAAUCCCCGCUAAUCGGGAGGCUGGAGGCAGGAGAAGCGCUCGAACCCGGGAGGCGGAAGGUGCGGUGAGCCAAGAUCGCGCCAUUGCACUCUAGCCGUGGAAACAAGAGUGAAACUCUGUCUCAAAAGGACGAAAC	
ABC DNA probe	GTTTCGTCCTTTTGAGACAGAGTTTCACTCTTGTTTCCACGGCTAGAGTGCAATGGCGCGATCTTGGCTCACCGCACCTTCCGCCTCCCGGGTTCGAGCGCTTCTCCTGCCTCCAGCCTCCCGATTAGCGGGGATTGACAGGGAGGCACCCCCACGCCTGGCTTGGCTGATGTTTGTGTTTTTAGTAGGCACGCCGTGTCTCTCCATGTTGCTCAGGCTGGTCTCCAACTCCCGACCTCCTGTGATGCGCCCACCTCGGCCTCTCGAAGTGCTGGGATGACGGGCGTGACGACCGTGCCCGGCCTGTTGACTCATTTCGCTTTTTTATTTCTTTCGTTTCCACGCGTTTACTTATATGTATTAATGTAAACGTTTCTGTACGCTTATATGCAAACAACGACAACGTGTATCTCTGCATTGAATACTCTTGCGTATGGTAAATACGTATCGGTTGTATGGAAATAGACTTCTGTATGATAGATGTAGGTGTCTGTGTTATACAAATAAATACACATCGCTCTATAAAGAAGGGATCGTCGATAAAGACGTTTATTTTACGTATGAAAAGCGTCGTATTTATGTGTGTAAATGAACCGAGCGTA	
